# Interactive impact of salinity and oxygen level on the growth performance, digestive enzymes, serum biochemistry, antioxidative, immunity, and histological status of Nile tilapia (*Oreochromis niloticus*)

**DOI:** 10.1007/s10695-025-01608-6

**Published:** 2025-12-08

**Authors:** Mohamed N. Monier, Sherien H. H. Shady, Youssif Shehata Grana, Haytham A. Abd El-Ghaffar, Fatma Samir, Suzan O. M. El-Werwary, Ahmed A. Ahmed, Mohsen Abdel-Tawwab

**Affiliations:** 1 Department of Fish Biology and Ecology, Central Laboratory for Aquaculture Research, Agricultural Research Center, Abbassa, Abo-Hammad, Sharqia, 44662 Egypt; 2 Limnology Department, Central Laboratory for Aquaculture Research, Agricultural Research Center, Abbassa, Abo-Hammad, Sharqia, 44662 Egypt; 3 Department of Hatchery and Fish Physiology, Central Laboratory for Aquaculture Research, Agricultural Research Center, Abbassa, Abo-Hammad, Sharqia, 44662 Egypt; 4 Department of Fish Nutrition, Central Laboratory for Aquaculture Research, Agricultural Research Center, Abbassa, Abo-Hammad, Sharqia, 44662 Egypt; 5 Department of Fish Health and Management, Central Laboratory for Aquaculture Research, Agricultural Research Center, Abbassa, Abo-Hammad, Sharqia, 44662 Egypt

**Keywords:** Climate change, Hypoxia, Salinity stress, Growth performance, Hematology, Freshwater fish, Enzymatic activity, Immunological response, Inner organ histopathology, Fish welfare

## Abstract

Climate change, including global warming, is associated with an increase in water temperature, which leads to increased water evaporation from water bodies, resulting in elevated salinity and decreased dissolved oxygen (DO_2_) levels. This may deteriorate fish health and productivity, and threaten the sustainability of aquaculture. Hence, the current study was carried out to investigate the interactions between hypoxia and increased salinity, as well as their impact on growth parameters, digestive enzymes, serum biochemistry, antioxidative response, and histopathology in Nile tilapia (*Oreochromis niloticus*). A total of 270 juvenile fish were impartially allocated into 18 aquariums (six treatments with three replicates) in a 2 × 3 factorial design, which included two oxygen levels (normoxia = 5.5–6 mg/L DO_2_ and hypoxia = 1–1.5 mg/L DO_2_) and three salinity conditions (0, 7, and 14 g/L) over 56 days feeding on a commercial diet (32% protein). Salinity and hypoxia significantly reduced growth and feed utilization. The most declared weight gain and feed conversion ratios were obtained under normoxic and freshwater conditions, although the survival rate was not considerably altered. Hypoxia increased RBCs, hemoglobin, and hematocrit, while elevated salinity significantly reduced them. Hypoxia and elevated salinity impaired digestive enzymes (protease, lipase, α-amylase), increased plasma cortisol, glucose, and liver enzyme levels (aspartate aminotransferase and alanine aminotransferase), lipid profile levels (total cholesterol and triglycerides), while decreasing plasma total protein. The immunity response (lysozyme activity, respiratory burst, phagocytosis, and IgM) was markedly reduced under hypoxia and hypersalinity, while they were markedly enhanced under normoxia and freshwater conditions. Fish reared under hypoxia and higher salinity exhibited structural damage in gills, intestine, and liver tissues. Our findings show that environmental stressors (hypoxia and excessive salinity) harm Nile tilapia growth and well-being, emphasizing the need to improve aquaculture settings in response to climate change.

## Introduction

Aquaculture is crucial for meeting the global demand for aquatic products. However, climate change threatens its sustainability (FAO 2020a) and is the main cause of instability in aquaculture (Zarantoniello et al. 2021), which affects the ecosystem’s temperature, oxygen saturation, and water salinity (Albaqami and Monier 2025; Dawood et al. 2022a). Fish farms mainly use rivers and lakes, although high summer temperatures have increased evaporation (Chang et al. 2021b; Dawood et al. 2022a) and simultaneously decrease dissolved oxygen levels by approximately 2.3% per 1 °C increase (Rajesh and Rehana 2022), which further stresses the aquatic environment. Thus, the water salinity has risen, lowering oxygen levels and affecting osmoregulation (Lassoued et al. 2023). Climate change alters the physiological, biochemical, and genetic features of aquaculture species, threatening their health and survival (Stocker 2014).

High salinity adversely affects growth patterns, fish biology, osmoregulation, physiological condition, health, and homeostasis, as well as immune responses in freshwater fish (Ahmed et al. 2023; Ouyang et al. 2023). Therefore, it must be upheld at optimal levels to promote appropriate growth, metabolism, and physiological responses (Cui et al. 2019; Dawood et al. 2021). Many aquatic organisms have specific salinity ranges for optimal health, and deviations from these ranges can cause mortality, impaired growth, and reduced immunity (Agarwal et al. 2024; Jahan et al. 2019). Euryhaline teleosts, which exhibit remarkable adaptability to various salinities, employ efficient osmoregulatory strategies to maintain homeostasis (Hwang et al. 2011). However, several studies have indicated that despite this adaptability, their optimal growth performance and wellbeing are generally observed at low salinity levels, whereas higher salinities impose additional osmoregulatory and metabolic costs (Kamal & Mair 2005; Imsland et al. 2008).

Dissolved oxygen (DO_2_) is crucial for fish viability and plays a critical function in their performance (Fan et al. 2020; Yang et al. 2021). When water temperature rises, it accelerates the release of DO_2_ to the atmosphere, reducing water levels in rearing water, causing hypoxia stress or even the death of farmed fish (Brander 2007; Yang et al. 2021). Generally, hypoxic stress is a fundamental aspect that affects the growth and survival of finfish species via deteriorating several biochemical and physiological functions (Abdel-Tawwab et al. 2019; Dawood et al. 2021; Li et al. 2017). It could also alter respiratory and osmoregulatory processes, heightening oxidative stress (Sun et al. 2020). Moreover, low DO_2_ decreases nutrient metabolism and feed digestibility, leading to growth retardation and physiological and immunological dysfunctions in aquatic organisms (Sheng et al. 2019).

Fish’s intestine, liver, and gills are essential for preserving physiological homeostasis (Buddington et al. 1997; Evans et al. 2005; Hinton et al. 2018). Although the intestine absorbs nutrients and provides immunological protection, hypoxia and hypersalinity may affect its barrier integrity, leading to inflammation and reduced digestive efficiency (Dawood et al. 2022c; Jiang et al. 2023). The liver, crucial for metabolism and detoxification, experiences oxidative stress and hepatocellular degeneration due to prolonged hypoxia and fluctuations in salinity, which undermines its functions in energy storage and immune regulation (Huang et al. 2021; Tseng and Hwang 2008). Likewise, gills, essential for respiration and ion exchange, experience structural damage, including lamellar fusion, epithelial lifting, and ionocyte proliferation when subjected to extreme salinity and oxygen depletion, which hinders gas exchange and osmoregulation (Abdel-Tawwab et al. 2019; Evans et al. 2005). These stressors disrupt normal physiological processes, heightening disease susceptibility and diminishing aquaculture productivity.

Nile tilapia (*Oreochromis niloticus*) is known as a “chicken of aquaculture” or “Golden fish” due to its high resistance to intensive systems and adaptation to changing circumstances and stressors (El-Sayed 2019; FAO 2020b) such as hypoxia (Li et al. 2018), ammonia accumulation (Hegazi et al. 2010), salinity (El-Leithy et al. 2019), and inappropriate water temperature (Xavier et al. 2020). Nile tilapia exhibited multiple behaviors when cultivated in varied water salinities, which were associated with fluctuations in other variables, for instance, temperature, oxygen availability, ammonia, and feeding strategies (Dawood et al. 2021; Durigon et al. 2020).

Optimizing aquaculture circumstances in the face of climate change-induced environmental changes requires an understanding of the interactive implications of hypoxia and salinity stress. Although earlier studies have investigated salinity and hypoxia, they have been studied separately in fish physiology, and few studies have evaluated their interacting effects on Nile tilapia growth, metabolism, immunity, and histopathology over time. This study addresses this knowledge gap. This study is novel in its comprehensive assessment of multiple physiological and biochemical parameters, including growth performance, digestive enzyme activity, immune responses, oxidative stress, and histological responses to salinity and hypoxia stress under controlled experimental conditions. It provides sustainable fish farming guidelines for variable aquatic conditions. This research contributes to the development of resilient aquaculture strategies by examining how Nile tilapia adapts to long-term hypoxia and fluctuations in salinity, providing critical insights into its stress resistance pathways.

## 2. Materials and methods

### Study design and experimental circumstance

**Fig. 1:**
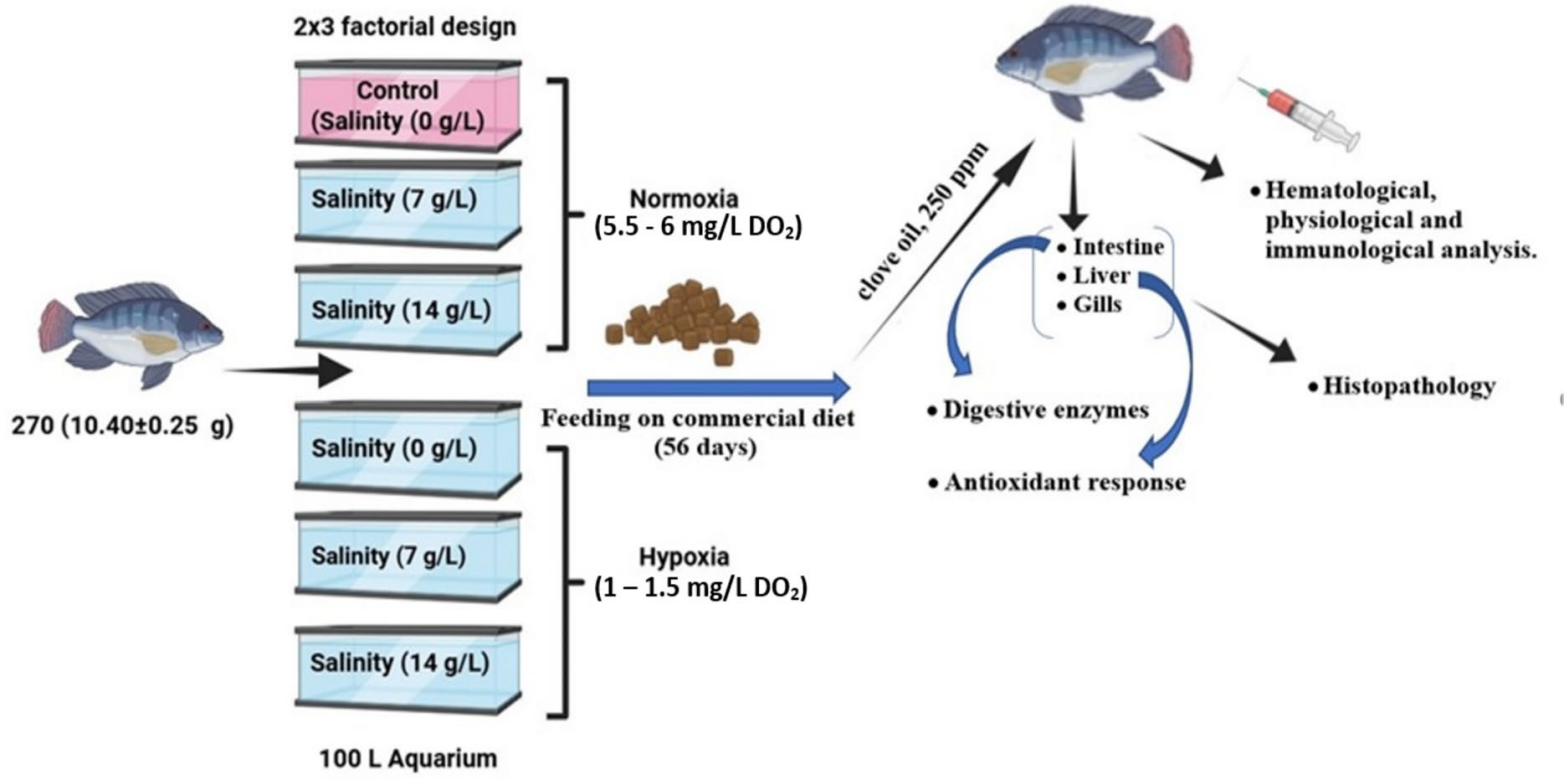
Experimental design schematic for Nile tilapia (*O. niloticus*) under varying salinity and oxygen levels. This 2 × 3 factorial design includes six treatment groups with three replicates each: three salinity levels (0, 7, and 14 g/L NaCl) crossed with two oxygen conditions (Normoxia = 5.5–6.0 mg/L DO_2_; Hypoxia = 1–1.5 mg/L DO_2_). Fish were fed a commercial diet for 56 days, and measurements included growth performance, digestive enzymes, antioxidant response, hematology, and histopathology

This investigation assessed the interactive impact of raising the water salinity and hypoxia on Nile tilapia (*O. niloticus*). A total of 270 juvenile mono-sex Nile tilapia (10.40 ± 0.25 g) were procured from WorldFish and acclimatized to experiment with wet lab conditions for 14 days in a rectangular plastic tank (2 m^3^). The Nile tilapia were randomly assigned to 18 100-L aquariums (15 fish/aquarium), representing six experimental groups with three replicas based on a 2 × 3 factorial design. The design, as illustrated in Fig. 1, has two oxygen levels (1–1.5 mg/L DO_2_, hypoxia, and 5.5–6 mg/L DO_2_, normoxia), and each has three salinity levels (0.0, 7, and 14 g/L). Water salinity was set by adding a commercial sea salt (Rich natural coarse sea salt, Seco Salt, Egypt) to the freshwater with the rates of 7 and 14 kg/m^3^ to get concentrations of 7 and 14 g/L, respectively, this mixture was used to more accurately represent the ionic composition of natural seawater and avoid the limitations of using NaCl alone and stored saline water and fresh water in three stock tanks (1000 L) for 24 h before introduction into the experimental tanks. Salinity in the trial aquarium was incrementally increased by adding sea salt to the water at a rate of 3‰ every day until the desired salinity levels were attained; thereafter, the experiment began. All fish were maintained under normal circumstances until the necessary water salinity was attained. Hypoxia was induced by regulating the aeration system to achieve targeted dissolved oxygen (DO) concentrations of 5–6 mg/L DO_2_ (normoxia) and 1–1.5 mg/L DO_2_ (hypoxia). Daily DO concentrations were monitored using a calibrated DO meter, and the aeration system was adjusted to ensure stable oxygen levels within these target ranges throughout the experiment. Fish were fed ad libitum twice every day at 9.00 and 14.00 h on a basal diet (32% crude protein, 6% crude lipid, Skretting, Bilbis, El Sharqia Governorate, Egypt) for 56 days.

Nile tilapia waste was removed daily, and two-thirds of aquarium water was changed with neat water from three reservoirs, each with a salinity corresponding to that used in the experiment. The experimental conditions were carefully monitored throughout the study, ensuring that water quality variables, including temperature, pH, and ammonia levels, remained within ideal limits for tilapia growth and survival (see Table 1 for optimal ranges). Salinity and dissolved oxygen were checked daily to maintain their levels within the specified ranges, allowing an accurate assessment of their effects on fish performance and health.

**Table 1:** Optimal water quality range according to Boyd and Tucker (2012)

Oxygen	Salinity levels (g/L)	Temperature (°C)	pH	Total ammonia (mg/L)	Free ammonia (mg/L)	Salinity (g/L)	TDS (g/L)	Conductivity (mS/cm)	DO_2_ (mg/L)
Optimal water quality range according to Boyd and Tucker (2012)		25–30 °C	6.5–8.5	< 0.5 mg/L	0.0–0.02 mg/L	0–14 g/L	0.2–1.0 g/L	0.2–1.5 mS/cm	> 5 mg/L

### Growth performance assessment

At the end of this trial, fish from each aquarium were harvested, counted, and group-weighed. Afterwards, growth performance and feed utilization were calculated using the subsequent standard formulas:

$$Weight\;gain\;(WG,g)=\:W_{final}(g)\:-W_{initial}(g),$$

$$Weight\;gain\;rate\;(WGR,\%)=\frac{W_{final}{(g)\;}-W_{initial}{(g)}}{W_{initial}{(g)}}\times100$$

$$The\;specific\;growth\;rate\;(SGR,\%/day)=\frac{ln\;W^{final}{\;(g)}\,-ln\;W^{initial}{\;(g)}}{t{\;(56days)}}\times100$$

$$Feed\;consumption\;(g\;feed/fish)\:=\:total\;amount\;of\;feed\;provided-\:the\;leftover\;feed,$$

$$feed\;conversion\;ratio\;(FCR)=\frac{Feed\;consumption\;(g\;)}{WG\;(g\;)}$$

$$Survival\;rate\;(SR,\%)=\frac{N^{final}}{N^{initial\,}}\times100$$

where *W*_final_ is the final mean body weight of fish (g), *W*_initial_ is the initial mean body weight of fish (g), *t* is the experiment period (56 days), *N*_final_ is the number of fish at the end of the experiment, and *N*_initial_ is the number of fish at the start of the experiment.

### Water quality measurements

Frequent water quality assessments were performed in all aquariums during the trial. Temperature and pH were taken via a portable HANA pH Tester (pH/ISE/ORP Meter, HANA, Finland). Dissolved oxygen was measured daily using a Jenway 970 DO_2_ meter (Jenway, London, UK). Salinity, conductivity, and TDS were determined by the HC3010 Conductivity Meter Portable (Trans Instruments, Singapore). Salinity was measured daily. The ammonia concentration was estimated thrice a week via the HACH test kit Ammonia Mid Range photometer (Nitrogen, Ammonia Test Kit, Model NI-SA, HACH Company, USA) according to Boyd and Tucker’s (1998).

### Sample gathering

At the end of the trial, the final body weight and total number of Nile tilapia were obtained after a 24-h fasting period. Eight fish were randomly chosen from each aquarium and submerged in an anesthetic liquid (clove oil, 250 ppm). Blood samples were obtained from the caudal vein with a heparinized needle. Fish were then dissected, and the mid portion of the intestine (about 1.0-cm long), liver, and gills were collected and divided into two sets, the first fixed in 4% paraformaldehyde for histopathology. At the same time, the second was placed in a plastic case and preserved in a freezer at − 21 °C for further digestive enzyme tests and antioxidant activity.

### Sample preparation for further measurements

Blood samples were divided into dual sets. The first sample used whole blood for hematological indices and respiratory burst activity (RBA), while the other was transferred into an Eppendorf tube. Plasma was split by a centrifuge (80–1 electric centrifuge, China) for 15 min at 4000 rpm. Plasma was kept at − 21 °C pending further physiological and immunological analysis.

Intestinal and hepatic tissues were promptly excised and homogenized using a chilled sucrose solution (0.25 M) utilizing a Swirlex – Digital Handheld Homogenizer (Germany) to create a 5% tissue homogenate. Tissue homogenates were subjected to centrifugation at 10,000 rpm for about 15 min at 4 °C using a centrifuge (TR16-M, TOMY, Japan), and the resultant supernatant was kept in tubes at − 21 °C pending digestion enzyme activity, lipid peroxidation (LPO), and antioxidant examination (Chong et al. 2002).

### Digestive enzyme activity and growth hormone

The protease concentration was evaluated using 2% Azocasein as a substrate in a Tris–HCl liquid at a pH of 7.5 (García-Carreño and Haard 1993). Lipase concentration was assessed using nitrophenyl myristate as a substrate, measured at 1 μmol of hydroxy nitrobenzene per mg of protein per minute (Iijima et al. 1998). The activity of α-Amylase was evaluated using starch as a substrate, according to the methodology outlined by Bernfeld (1955). The maltose production rate was measured at 1 μmol per milligram of protein per minute. Growth hormone quantities in ng/mL were measured via commercial Growth Hormone ELISA Kit (EHGH1, Thermo Fisher Scientific, Waltham, MA, USA), following the manufacturer’s guidelines.

### Lipid peroxidation and antioxidant activity

Total protein was assessed via the technique established by Lowry et al. (1951), employing Folin’s reagent and bovine serum albumin (BSA) as a standard. LPO quantities were assessed using the malondialdehyde (MDA) concentration resulting from the calibration curve (Uchiyama and Mihara 1978). The absorbance of the supernatant was determined at 520 and 535 nm. The concentration of MDA was quantified as nmol MDA per mg of protein.

The SOD activity in the tissue extract at 550 nm absorbance was evaluated using the pyrogallol auto-oxidation technique induced by superoxide radicals and reported as U/mg protein (Marklund and Marklund 1974). The reduction in absorbance of H_2_O_2_ at 240 nm was used to assess catalase (CAT) activity and was expressed as µmol/mg protein/min (Aebi 1984). The glutathione peroxidase (GPx) activity was measured by assessing the oxidation of nicotinamide adenine dinucleotide phosphate (NADPH) consumed at 340 nm using t-butyl hydroperoxide as a substrate (Flohé and Günzler 1984). The activity of GPX was quantified as U/mg protein. All experiments were performed at 25 ± 0.5 °C using a spectrometer (Lambda 2S UV, PerkinElmer Co., USA).

### Hematological indices

The quantities of red blood cells (RBCs) were assessed using Neubauer’s hemocytometer, following the technique mentioned by Hendricks (1952). White blood cell (WBCs) quantities were estimated according to Lewis et al. (2006). The hemoglobin (Hb) concentration was assessed via the spectrophotometric cyanmethemoglobin technique (van Kampen and Zijlstra 1961). The hematocrit (HCT) was assessed in microhematocrit-heparinized capillaries within 40-min post-blood sampling, utilizing a microhematocrit centrifuge at 13,000 rpm for 3 min (Briggs and Bain 2017). The evaluation of derived erythrocyte parameters, such as mean corpuscular volume (MCV), mean corpuscular hemoglobin (MCH), and mean corpuscular hemoglobin concentration (MCHC), was conducted employing the equations established by Telli et al. (2014) as follows:

$$MCV\;(fL)=\frac{Hematocrite\;\left(\%\right)\times10}{RBCs\;count\;(10^6/\mu L)}$$

$$MCH\;(pg)=\frac{Hemoglobin\;(g/dL)\times10}{RBCs\;count\;(10^6/\mu L)}$$

$$MCHC\;(g/dL)=\frac{Hemoglobin\;(g/dL)\times100}{Hematocrite\;\left(\%\right)}$$

### Biochemical analysis

Plasma cortisol concentrations were measured using radioimmunoassay kits sourced from Bayer, following the assay, chemicals, and methods outlined by Pankhurst and Sharples (1992). Commercial colorimetric assay kits from Diamond, Egypt, were used to measure plasma glucose (Trinder 1969), aspartate aminotransferase (AST) and alanine aminotransferase (ALT) (Reitman and Frankel 1957), total protein (Doumas 1975), and albumin (Doumas et al. 1971). The equations used for calculating plasma globulin are as follows:

$$Globulin\;(g/dL)=Total\;protein\;(g/dL)\:-Total\;albumin\;(g/dL).$$

Commercial assay kits from Biodiagnostic Co., Egypt, were used to assess total cholesterol (T-CHO) (Richmond 1973) and triglycerides (TG) (Fossati and Prencipe 1982).

### Histological examination of the intestine, liver, and gills

The mid-part of the intestinal tract, liver, and gills were preserved in 4% paraformaldehyde for more than 24 h, adhering to standard histological techniques. Following fixation, tissue samples underwent dehydration in ethanol with serial dilution, were cleared with xylene, and subsequently fixed in paraffin blocks (Bancroft 2008). Subsequently, 4-μm-thick tissue segments were prepared through a semi-automated rotary microtome (M-240, MYR, Spain). Fifty percent of these slices were dyed using Haematoxylin and Eosin (H and E) following the procedure outlined by Suvarna et al. (2012). Observations were conducted using Meiji Techno MT4500 Series with a Digital Camera microscope (Meiji Techno Co., Ltd., Japan) and analyzed using Meiji’s MDS-800 software. The villus height, number, mucosal fold width, lamina propria thickness, and intestinal wall thickness in the intestine were examined and determined with the CaseViewer application. Histopathological scores were identified for the intestine, gills, and liver following Treuting and Boyd (2019). Histopathological scores were identified for the intestine, gills, and liver tissues. The degrees of lesions were referred to as negative (−), weak (+), moderate (+ +), and severe (+ + +). Measurements were conducted in micrometers (μm), and the data were statistically evaluated.

### Immunity indicators

Serum lysozyme activities were turbidimetrically examined through *Micrococcus luteus* as a substrate (Ellis 1990), defined as the quantity of enzyme necessary to drop the absorbance value by 0.001/min in 1 mL of the animal’s serum.

Respiratory burst activity (RBA) was quantified by introducing 50 µL of blood into the wells of a microplate, followed by adding 50 µL of a 0.2% nitroblue tetrazolium (NBT) solution to each well. The plate was then left to be incubated for 30 min at room temperature. Fifty microliters of the NBT-blood cell suspension was then combined with 1 mL of N,N-dimethylformamide (Sigma) and centrifuged for 5 min at 3000 rpm. The supernatant’s absorbance was determined using a spectrophotometer at 620 nm (Rook et al. 1985).

The methodology of Kawahara et al. (1991) was applied to evaluate phagocytic activity (PA) by adding 0.1 mL of blood to a dish and thoroughly stirring it with 25 mL of *Candida* sp. for 2 h at ambient temperature. Subsequently, it was fixed with 100% methanol for about 5 min and stained with 10% Giemsa (Merck, Germany) for 15 min. After removing excess dye using phosphate-buffered saline (PBS, pH 7.4), the number of phagocyte cells per 300 adherent cells was enumerated microscopically. The number of cells that had engulfed at least one particle was counted, and the percent of phagocytic cells was determined through the following equation:

$$Phagocytic\;Activity=\frac{Number\;of\;Phagocytic\;Cells}{The\;total\;number\;of\;cells\;observed}\times100$$

The phagocytic index was calculated by determining the number of particles each phagocytic cell engulfed. Following the assessment of phagocytic cell quantity, the aggregate number of particles internalized by these cells was enumerated. The phagocytic index was calculated via the following equation:

$$Phagocytic\;Index=\frac{The\;total\;number\;of\;phagocytic\;cells\;observed}{Total\;number\;of\;particles\;engulfed}$$

Immunoglobulin M (IgM) was assessed using the techniques described by Siwicki (1993).

### Statistical analysis

Levene’s test was employed to evaluate the homogeneity of variance within the data. Normality of the dataset was tested using the Shapiro–Wilk test. Data that met the assumptions of normality and homogeneity were analyzed using one-way and two-way ANOVA. When significant effects were detected, Duncan’s multiple-range test was used as a post hoc test for all pairwise comparisons among treatments. Otherwise, the data were investigated using Tamhane’s T2 test. Every record was examined using one-way analysis of variance (ANOVA) in IBM SPSS version 26.0 (IBM SPSS Inc., Chicago, USA). Alterations were deemed statistically substantial at *P* < 0.05. For clarity, letters indicating significant differences (*P* < 0.05) were displayed only for parameters showing significant variation; parameters without significant differences were presented without letters, as identical letters would indicate homogeneity. When significant differences were found, the influence of water salinity, hypoxia, and their interaction on Nile tilapia performance was assessed via two-way ANOVA.

## Results

### Water quality parameters

Water quality measurements were acceptable and were not substantially affected by hypoxia, salinity, or their interaction (*P* > 0.05; Table 2). Only the salinity-related variables, such as TDS and conductivity, were affected by salinity level and increased with salinity. All treatments displayed ideal water temperatures, pH levels, total ammonia, and free ammonia concentrations without substantial variance (*P* > 0.05; Table 2) between all groups.

**Table 2:** The aquarium’s water quality was reared with Nile tilapia (*O. niloticus*), which was stressed by hypoxia and salinity for 56 days

Oxygen	Salinity levels (g/L)	Temperature (°C)	pH	Total ammonia (mg/L)	Free ammonia (mg/L)	Salinity (g/L)	TDS (g/L)	Conductivity (mS/cm)	DO_2_ (mg/L)
	Freshwater (HS0)	25.43 ± 0.03	7.43 ± 0.03	1.23 ± 0.05	0.019 ± 0.001	0.20 ± 0.00 c	0.176 ± 0.001 c	0.353 ± 0.002 c	1.11 ± 0.20 b
Hypoxia (H)	7.0 (HS7)	25.43 ± 0.03	7.47 ± 0.03	1.41 ± 0.22	0.021 ± 0.003	7.03 ± 0.03 b	6.64 ± 0.06 b	12.987 ± 0.027 b	1.12 ± 0.10 b
	14.0 (HS14)	25.47 ± 0.03	7.47 ± 0.03	1.15 ± 0.05	0.017 ± 0.001	13.97 ± 0.23 a	12.11 ± 0.20 a	23.397 ± 0.003 a	1.27 ± 0.19 b
	Freshwater (NS0)	25.47 ± 0.03	7.47 ± 0.03	1.60 ± 0.23	0.024 ± 0.004	0.20 ± 0.00 c	0.16 ± 0.01 c	0.357 ± 0.001 c	5.83 ± 0.18 a
Normoxia (N)	7.0 (NS7)	25.43 ± 0.03	7.43 ± 0.03	1.48 ± 0.19	0.022 ± 0.003	7.10 ± 0.15 b	6.58 ± 0.14 b	13.17 ± 0.28 b	5.88 ± 0.19 a
	14.0 (NS14)	25.43 ± 0.03	7.43 ± 0.03	1.51 ± 0.18	0.023 ± 0.003	14.07 ± 0.09 a	12.16 ± 0.09 a	23.37 ± 0.03 a	5.80 ± 0.21 a
Two-way ANOVA		*P* value							
Oxygen		1.000	0.690	0.080	0.081	0.582	0.942	0.603	0.0001
Salinity		0.848	1.000	0.781	0.794	0.0001	0.0001	0.0001	0.943
Oxygen × salinity		0.619	0.531	0.628	0.678	0.915	0.869	0.639	0.796

Mean values ± standard error followed by different letters in the same column are substantially different at *P* < 0.05

### Growth performance and feed utilization

Growth performance and feed consumption were substantially influenced by hypoxia and salinity and their interaction (*P* < 0.05; Table 3). Final weight, weight gain, SGR, and feed consumption substantially decreased with dissolved oxygen, salinity, and their interaction (*P* < 0.05). FCR values were substantially (*P* < 0.05) reduced at low salinity with normoxia (*P* < 0.05), and the optimal FCR was at normoxia treatment with freshwater (control; NS0; 1.56), while the highest value was at the interaction between hypoxia and salinity 14 g/L (HS14; 2.29; Table 3). The fish survival ratio is unaffected by increased salinity, hypoxia, or interaction (*P* > 0.05).

**Table 3:** Growth performance and feed utilization of Nile tilapia (*O. niloticus*) stressed by hypoxia and salinity levels for 56 days

Oxygen	Salinity levels (g/L)	Initial weight (g)	Final weight (g)	Weight gain (g)	Weight gain % (%)	SGR (%/day)	Feed consumption (g feed/fish)	FCR	Fish survival (%)
	Freshwater (HS0)	10.43 ± 0.24	22.17 ± 0.18 d	11.73 ± 0.29 d	112.68 ± 4.99 d	1.35 ± 0.04 d	23.47 ± 0.49 d	2.00 ± 0.01 b	97.78 ± 2.22
Hypoxia (H)	7.0 (HS7)	10.27 ± 0.09	18.60 ± 0.21 e	8.33 ± 0.28 e	81.22 ± 3.45 e	1.06 ± 0.03 e	18.07 ± 0.23 e	2.17 ± 0.05 a	97.78 ± 2.22
	14.0 (HS14)	10.20 ± 0.20	15.80 ± 0.31 f	5.60 ± 0.12 f	54.9 ± 0.58 ef	0.78 ± 0.006 f	12.8 ± 0.31 f	2.29 ± 0.04 a	95.56 ± 2.22
	Freshwater (NS0)	10.43 ± 0.24	36.53 ± 0.43 a	26.10 ± 0.67 a	250.71 ± 11.95 a	2.24 ± 0.06 a	40.47 ± 1.17 a	1.56 ± 0.07 d	100.00 ± 0.00
Normoxia (N)	7.0 (NS7)	10.40 ± 0.25	31.17 ± 0.85 b	20.77 ± 1.07 b	200.36 ± 14.57 b	1.96 ± 0.09 b	35.90 ± 1.02 b	1.73 ± 0.05 c	97.78 ± 2.22
	14.0 (NS14)	10.50 ± 0.17	26.57 ± 0.67 c	16.07 ± 0.50 c	152.95 ± 2.46 c	1.66 ± 0.02 c	30.23 ± 0.38 c	1.88 ± 0.04 b	97.78 ± 2.22
Two-way ANOVA		*P* value							
Oxygen		0.409	< 0.0001	< 0.0001	< 0.0001	< 0.0001	< 0.0001	< 0.0001	0.389
Salinity		0.876	< 0.0001	< 0.0001	< 0.0001	< 0.0001	< 0.0001	< 0.0001	0.564
Oxygen × salinity		0.772	0.013	0.019	0.087	0.018	0.040	0.881	0.821

Mean values ± standard error followed by different letters in the same column are substantially different at *P* < 0.05

### Digestive enzyme secretion and growth hormone

**Fig. 2:**
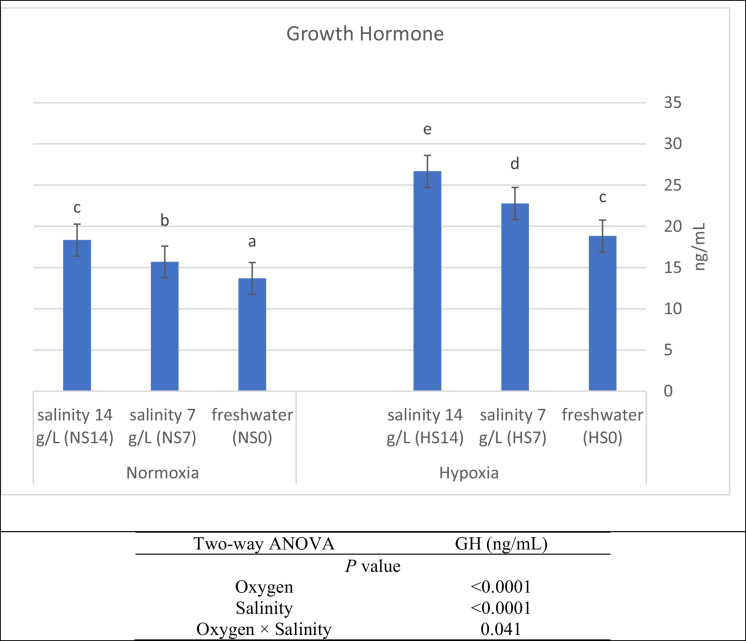
Activities of growth hormone of Nile tilapia (*O. niloticus*) stressed by hypoxia and salinity levels for 56 days. Parameters with the same letter are not substantially different, while those with different letters are substantially different (*P* < 0.05)

**Fig. 3:**
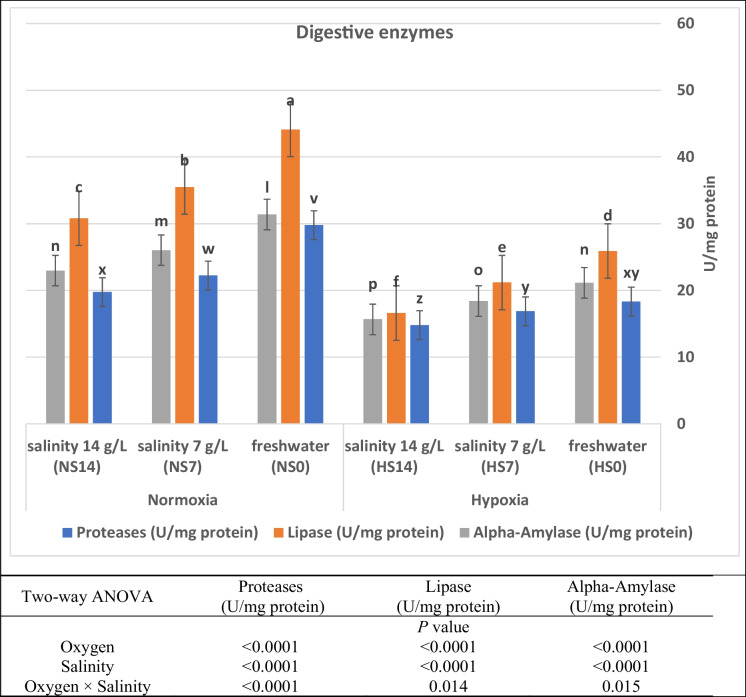
Activities of intestinal digestive enzymes of Nile tilapia (*O. niloticus*) stressed by hypoxia and salinity levels for 56 days. Alpha-Amylase: Labeled with mixed case letters (l, m, n, o, p); Lipase: Labeled with small letters (a, b, c, …); Proteases: Labeled with capital letters (v, w, x, y, z). Parameters with the same letter are not substantially different (*P* > 0.05), while those with different letters are substantially different (*P* < 0.05)

The growth hormones and digestive enzyme secretions were substantially (*P* < 0.05) affected by salinity, DO_2_, and their interaction (Figs. 2 and 3). Protease, lipase, α-amylase, and growth hormones decreased substantially (*P* < 0.05) with increased salinity under different DO_2_ concentrations. Moreover, under hypoxic conditions, this decrease in enzyme secretion was more pronounced, as the group with anoxia and higher salinity exhibited lower levels of the enzymes. The better values for digestive enzymes and growth hormone were at normoxic treatment with freshwater (NS0) (Fig. 3).

### Blood profile indices

Hematological parameters of Nile tilapia stressed by hypoxia and salinity were substantially impacted by salinity, dissolved oxygen, and their interaction (*P* < 0.05; Table 4). RBCs, hemoglobin, and hematocrit were generally higher under hypoxic conditions compared to normoxia at a given salinity, although they declined with increasing salinity within each oxygen level. WBCs counts increased under both hypoxic and high-salinity conditions. The highest hematology readings were for normoxic treatment with freshwater (NS0), while the lowest values were for the interaction between hypoxia and salinity, especially for the treatment with salinity 14 g/L (HS14). On the other hand, MCV, MCH, and MCHC were not influenced (*P* > 0.05) by DO_2_, salinity, or their interaction (Table 4).

**Table 4:** Changes in hematological indices of Nile tilapia (*O. niloticus*) stressed by hypoxia and salinity levels for 60 days

Oxygen	Salinity levels (g/L)	WBCs (× 10^3^ μL)	RBCs(× 10^6^ μL)	Hemoglobin (g/dL)	Hematocrit (%)	MCV (fL)	MCH (pg)	MCHC (g/dL)
	Freshwater (HS0)	11.51 ± 0.25 c	4.58 ± 0.17 a	14.17 ± 0.12 a	51.00 ± 1.73 a	111.90 ± 6.87	31.07 ± 1.35	27.84 ± 0.73
Hypoxia (H)	7.0 (HS7)	12.53 ± 0.32 b	4.15 ± 0.12 b	12.52 ± 0.62 b	45.67 ± 1.86 b	110.36 ± 6.70	30.16 ± 1.15	27.48 ± 1.49
	14.0 (HS14)	13.84 ± 0.42 a	3.79 ± 0.08 bc	11.91 ± 0.14 bc	41.33 ± 1.20 c	109.37 ± 5.32	31.48 ± 0.99	28.84 ± 0.52
Normoxia (N)	Freshwater (NS0)	9.20 ± 0.21 e	3.72 ± 0.14 c	11.84 ± 0.11 bc	41.00 ± 1.15 c	110.73 ± 6.09	31.95 ± 1.40	28.93 ± 0.99
	7.0 (NS7)	10.33 ± 0.33 d	3.31 ± 0.06 d	10.99 ± 0.12 c	36.67 ± 0.33 d	110.82 ± 1.65	33.22 ± 0.69	29.97 ± 0.18
	14.0 (NS14)	11.45 ± 0.18 c	2.92 ± 0.11 e	9.44 ± 0.40 d	32.00 ± 0.58 e	109.71 ± 3.58	32.29 ± 0.60	29.48 ± 0.87
Two-way ANOVA		*P* value						
Oxygen		< 0.0001	< 0.0001	< 0.0001	< 0.0001	0.978	0.096	0.077
Salinity		< 0.0001	< 0.0001	< 0.0001	< 0.0001	0.946	0.941	0.691
Oxygen × salinity		0.043	0.042	0.031	0.023	0.986	0.511	0.571

Mean values ± standard error followed by different letters in the same column are substantially different at *P* < 0.05

### Physiological response

Dissolved oxygen and salinity and their interaction significantly impacted plasma cortisol, glucose, lipid profiles (total cholesterol, triglycerides), AST, and ALT of Nile tilapia (*P* < 0.05; Table 5). All the aforementioned indices substantially increased (*P* < 0.05) with rising salinity and continued to augment with hypoxia. The lowest values for the abovementioned variables were at normoxic treatment with freshwater (control; NS0), while the highest value was at hypoxia treatments with salinity 14 g/L (HS14).

**Table 5:** Changes in the levels of plasma cortisol, glucose, total cholesterol (T-CHO), triglycerides (TG), aspartate aminotransferase (AST), and alanine aminotransferase (ALT) of Nile tilapia (*O. niloticus*) stressed by hypoxia and salinity for 56 days

Oxygen	Salinity levels (g/L)	Cortisol (ng/dL)	Glucose (mg/dL)	Total cholesterol (mg/dL)	Triglyceride (mg/dL)	AST (U/L)	ALT (U/L)
	Freshwater (HS0)	28.73 ± 0.84 c	93.35 ± 0.52 c	89.85 ± 0.74 c	86.58 ± 0.99 c	32.59 ± 1.10 c	34.85 ± 0.34 c
Hypoxia (H)	7.0 (HS7)	32.81 ± 0.35 b	101.27 ± 2.18 b	95.07 ± 1.05 b	90.90 ± 0.95 b	38.54 ± 2.05 b	38.08 ± 0.30 b
	14.0 (HS14)	38.23 ± 0.93 a	117.97 ± 4.38 a	100.53 ± 1.11 a	96.87 ± 0.90 a	46.73 ± 1.73 a	42.60 ± 0.54 a
Normoxia (N)	Freshwater (NS0)	18.64 ± 1.15 e	76.07 ± 1.22 d	78.43 ± 1.58 e	76.29 ± 1.53 e	19.23 ± 0.44 e	25.20 ± 1.00 e
	7.0 (NS7)	23.19 ± 1.94 d	84.50 ± 0.53 e	85.33 ± 0.54 d	81.85 ± 1.00 d	23.34 ± 1.04 d	29.52 ± 0.61 d
	14.0 (NS14)	28.20 ± 1.14 c	92.83 ± 0.90 c	89.78 ± 1.32 c	85.98 ± 0.89 c	31.03 ± 0.90 c	34.47 ± 0.67 c
Two-way ANOVA		*P* value					
Oxygen		< 0.0001	< 0.0001	< 0.0001	< 0.0001	< 0.0001	< 0.0001
Salinity		< 0.0001	< 0.0001	< 0.0001	< 0.0001	< 0.0001	< 0.0001
Oxygen × salinity		0.006	0.017	0.012	0.028	0.037	0.042

Mean values ± standard error followed by different letters in the same column are substantially different at *P* < 0.05

### Protein profile

Salinity and dissolved oxygen significantly affected globulin, albumin, and total protein (*P* < 0.05; Table 6). However, total protein was substantially impacted by the interaction between salinity and dissolved oxygen (*P* < 0.05). Their levels were meaningfully reduced by increased salinity and continuously dropped by hypoxia. Furthermore, the highest levels for the aforementioned indices were observed in normoxia at freshwater (control; NS0), while the lowest occurred under hypoxia at 14 g/L salinity (HS14).

**Table 6:** Changes in protein profile (total protein, albumin, and globulin) of Nile tilapia (*O. niloticus*) stressed by hypoxia and salinity for 56 days

Oxygen	Salinity levels (g/L)	Total protein (g/dL)	Albumin (g/dL)	Globulin (g/dL)
	Freshwater (HS0)	3.10 ± 0.06 c	0.95 ± 0.08 bc	2.15 ± 0.09 b
Hypoxia (H)	7.0 (HS7)	2.83 ± 0.08 d	0.86 ± 0.09 bc	1.97 ± 0.02 bc
	14.0 (HS14)	2.57 ± 0.04 e	0.75 ± 0.07 c	1.82 ± 0.04 c
	Freshwater (NS0)	4.14 ± 0.10 a	1.36 ± 0.04 a	2.77 ± 0.10 a
Normoxia (N)	7.0 (NS7)	3.73 ± 0.12 b	1.05 ± 0.13 b	2.68 ± 0.02 a
	14.0 (NS14)	3.19 ± 0.06 c	1.09 ± 0.08 b	2.10 ± 0.06 b
Two-way ANOVA				
Oxygen		< 0.0001	< 0.0001	< 0.0001
Salinity		< 0.0001	0.034	< 0.0001
Oxygen × salinity		0.042	0.449	0.011

Mean values ± standard error followed by different letters in the same column are substantially different at *P* < 0.05

### Immune responses

The activities of lysozyme, respiratory burst, phagocytosis, and total IgM of Nile tilapia were substantially impacted by dissolved oxygen, salinity, and their interaction (*P* < 0.05; Table 7). These immunological parameters were substantially increased by decreasing salinity at normoxia. Therefore, the highest values were observed at normoxic treatment in freshwater (NS0), while the lowest values were recorded at treatment under hypoxia and salinity of 14 g/L (HS14). All the abovementioned indicators followed similar trends, decreasing under stress conditions (*P* < 0.05).

**Table 7:** Changes in the levels of blood lysozyme, respiratory burst activity (RBA), phagocytic activity, and total immunoglobulin (total IgM) of Nile tilapia (*O. niloticus*) were stressed by hypoxia and salinity for 56 days

Oxygen	Salinity levels (g/L)	Lysozyme (µg/mL)		RBA (mg/mL)		Phagocytic activity (%)		Phagocytic Index		Total IgM (mg/mL)	
	Freshwater (HS0)	5.53 ± 0.13 cd		0.95 ± 0.01 c		8.83 ± 0.08 c		0.94 ± 0.01 c		4.65 ± 0.09 c	
Hypoxia (H)	7.0 (HS7)	4.93 ± 0.09 de		0.86 ± 0.03 cd		7.24 ± 0.18 d		0.77 ± 0.02 d		4.11 ± 0.09 cd	
	14.0 (HS14)	4.40 ± 0.10 e		0.78 ± 0.02 d		6.19 ± 0.16 e		0.66 ± 0.02 e		3.70 ± 0.12 d	
	Freshwater (NS0)	9.12 ± 0.16 a		1.41 ± 0.09 a		11.94 ± 0.23 a		1.27 ± 0.03 a		7.55 ± 0.51 a	
Normoxia (N)	7.0 (NS7)	7.00 ± 0.44 b		1.13 ± 0.05 b		10.50 ± 0.29 b		1.12 ± 0.03 b		5.89 ± 0.12 b	
	14.0 (NS14)	5.93 ± 0.09 c		1.00 ± 0.02 bc		8.94 ± 0.37 c		0.95 ± 0.04 c		4.78 ± 0.14 c	
Two-way ANOVA			*P* value								
Oxygen			< 0.0001		< 0.0001		< 0.0001		< 0.0001		< 0.0001
Salinity			< 0.0001		< 0.0001		< 0.0001		< 0.0001		< 0.0001
Oxygen × salinity			0.001		0.017		0.015		0.025		0.006

Mean values ± standard error followed by different letters in the same column are substantially different at *P* < 0.05

### Lipid peroxidation and antioxidant activity

**Fig. 4:**
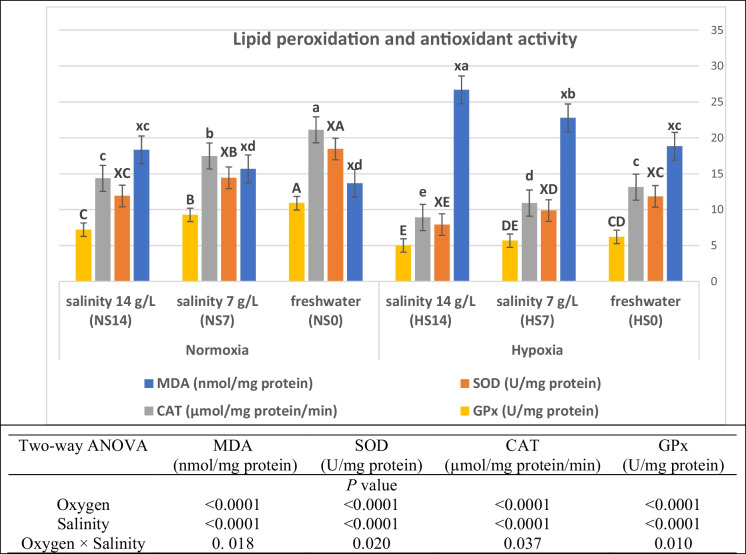
Changes in hepatic malondialdehyde (MDA), superoxide dismutase (SOD), catalase (CAT), and glutathione peroxidase (GPx) of Nile tilapia (*O. niloticus*) stressed by hypoxia and salinity for 56 days. GPx: Labeled with capital letters (A, B, C, …); CAT: Labeled with small letters (a, b, c, …); SOD: Labeled with mixed case letters (XA, XB, XC, …); MDA: Labeled with mixed case letters (xa, xb, xc, …). Parameters with the same letter are not substantially different (*P* > 0.05), while those with different letters are substantially different (*P* < 0.05)

Lipid peroxidation (malondialdehyde; MDA) and antioxidant activity are substantially impacted by dissolved oxygen and salinity stress and their interaction (*P* < 0.05; Fig. 4). MDA was significantly increased with stress by hypoxia and salinity, while SOD, CAT, and GPx of Nile tilapia were substantially decreased (*P* < 0.05). The lower values for lipid peroxidation (MDA) and the highest oxidative defense activity were at normoxic treatment at freshwater (control; NS0), while the highest MDA and the lowest antioxidant response were at hypoxia and salinity 14 g/L (HS14).

### Histopathology for the intestine, gills, and liver

The histopathological examination of *O. niloticus* intestine, hepatopancreas, and gills was prominently influenced by oxygen as well as salinity levels, as follows:

a The intestine:
  The intestine of the control group (NS0) displayed a normal, intact intestinal wall and intact intestinal villi, with a typical arrangement comprising four distinct layers: tunica mucosa, propria submucosa, tunica muscularis, and outer serosa (Fig. 5A). Morphometric analysis revealed that villus height and width, lamina propria thickness, intestinal wall thickness, and goblet cell number were all substantially influenced by hypoxia, salinity, and their interaction (Table 8). Under normoxic conditions, increased salinity levels resulted in intact but shorter villi with decreased branching, alongside increased villus width and lamina propria thickness (Fig. 5B, C).
  **Fig. 5:**
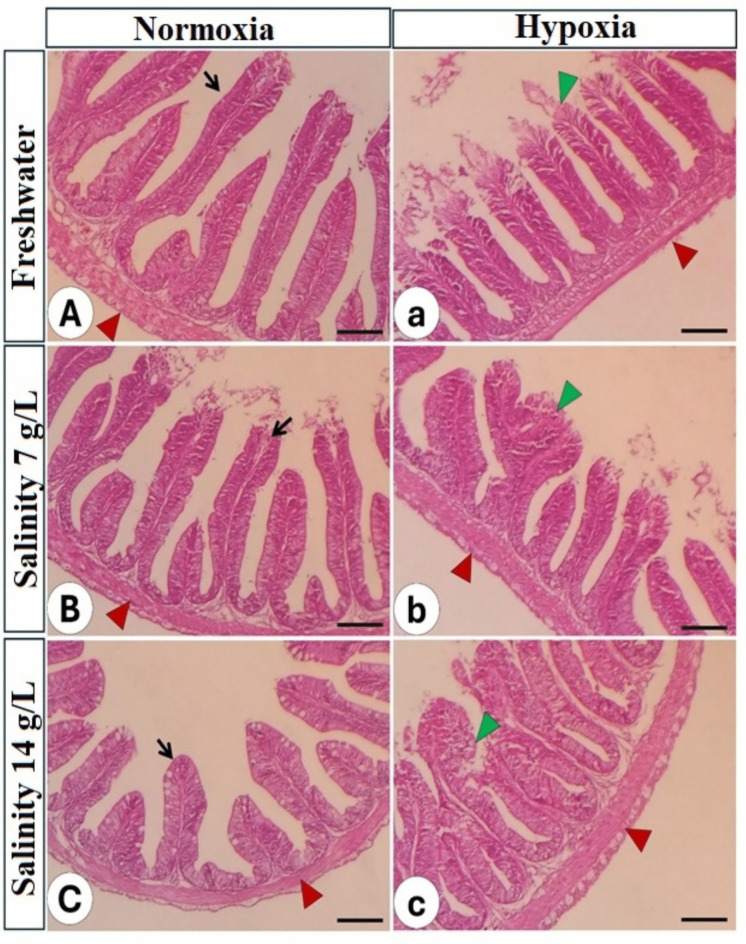
Histopathology of the middle segment of the intestine in Nile tilapia reared in different water salinity and two oxygen levels. The left column refers to normal oxygen and different salinity levels (A–C), while the right column refers to low oxygen and different water salinity (a–c). Black arrow: normal intestinal villi, green arrowhead: degeneration and sloughing of the lining epithelium of intestinal villi, red arrowhead: intestinal wall. Stain H&E. Bar = 100 µm
  **Table 8:** Changes intestinal morphpmetric parameters of Nile tilapia (*O. niloticus*) stressed by hypoxia and salinity for 56 days

Oxygen	Salinity levels (g/L)	Villus height (µm)	Villus width (µm)	Lamina propria thickness (µm)	Intestinal wall thickness (µm)	Goblet cell number
	Freshwater (HS0)	291.52 ± 2.97 ^c^	71.84 ± 2.18 ^d^	44.79 ± 0.51 ^bc^	55.85 ± 0.84 ^b^	8.67 ± 0.33 ^bc^
Hypoxia (H)	7.0 (HS7)	272.12 ± 6.05 cd	93.64 ± 3.70 ^ab^	47.62 ± 0.59 ^b^	68.84 ± 1.64 ^a^	9.67 ± 0.33 ^b^
	14.0 (HS14)	234.63 ± 10.0 ^d^	100.40 ± 3.14 ^a^	53.45 ± 1.63 ^a^	65.06 ± 2.46 ^a^	11.33 ± 0.33 ^a^
	Freshwater (NS0)	436.01 ± 25.75 ^a^	69.10 ± 4.29 ^d^	37.72 ± 1.03 ^e^	66.81 ± 2.20 ^a^	5.00 ± 0.58 ^e^
Normoxia (N)	7.0 (NS7)	339.92 ± 8.79 ^b^	81.27 ± 1.33 ^c^	40.86 ± 1.06 ^d^	55.00 ± 1.12 ^b^	6.67 ± 0.33 ^d^
	14.0 (NS14)	259.81 ± 3.93 cd	88.01 ± 0.90 ^bc^	44.40 ± 1.25 ^c^	46.69 ± 1.02 ^c^	7.67 ± 0.33 cd
Two-way ANOVA						
Oxygen		< 0.0001	0.002	< 0.0001	< 0.0001	< 0.0001
Salinity		< 0.0001	< 0.0001	< 0.0001	0.006	< 0.0001
Oxygen × salinity		0.001	0.019	0.043	< 0.0001	0.019

Mean values ± standard error followed by different letters in the same column are substantially different at *P* < 0.05
  Under hypoxic conditions, a more pronounced deterioration was observed. This was characterized by decreased villus height and branching, sloughing of the apical intestinal epithelium, and degeneration of enterocytes (Fig. 5a). Furthermore, at high salinity levels under hypoxia, there was marked hyperplasia of intestinal villi, intestinal wall thickening, and a significant increase in goblet cell number (Fig. 5b, c; Table 9).
  **Table 9:** Summary of the pathological lesions score in the intestine, gills, and liver of Nile tilapia (*O. niloticus*) stressed by hypoxia and salinity for 56 days

Organ	Lesions	Normoxia			Hypoxia		
		Salinity levels (g/L)			Salinity levels (g/L)		
		**0**	**7**	**14**	**0**	**7**	**14**
**Intestine**	Number of villi	+ + +	+ + +	+ + +	+ + +	+ +	+
	Branching of villi	+ + +	+ +	−	−	−	−
	Hyperplasia	−	+	+ +	−	+ + +	+ + +
	Degeneration and sloughing	−	−	−	+ +	+ + +	+ + +
**Gills**	Telangiectasia	−	−	−	** + **	** + + **	** + + + **
	Vascular congestion	−	−	** + + **	** + + **	** + + **	** + + + **
	Leukocytic infiltration	−	** + **	** + **	** + + **	** + + **	** + + + **
	Degeneration	−	** + **	** + **	** + + **	** + + + **	** + + + **
**Liver**	Vascular congestion	−	** + + **	** + + **	** + + **	** + + **	** + + **
	Nuclear pyknosis	−	−	−	−	** + **	** + + + **
	Vacuolation	−	−	−	−	** + + **	** + + + **
	Degeneration	−	−	−	−	** + + **	** + + + **
The degrees of lesions were referred to as negative (−), weak (+), moderate (+ +), and severe (+ + +). The pathological alteration increased in the Nile tilapia, which was subjected to double stress due to low oxygen and high salinity
b The gills:
  Histopathology of Nile tilapia gills reared in freshwater and at normal oxygen levels revealed intact epithelium of both primary and secondary filaments (Fig. 6A). Increased salinity level at salinity 7 g/L at normoxic condition triggered slight degeneration of the primary filament (Fig. 6B), in addition to primary vascular congestion at salinity 14 g/L (Fig. 6C; Table 9). At hypoxia, the hazard effects increased gradually with increased salinity. These changes include congestion of the primary filaments’ blood vessels, an increased count of chloride cells, dilatation of the apical part of the secondary filaments (telangiectasia) (Fig. 6a, b), and degeneration of the lining epithelium was observed in fish exposed to the combined stress of hypoxia and the highest salinity level of 14 g/L (Fig. 6c; Table 9).
  **Fig. 6:**
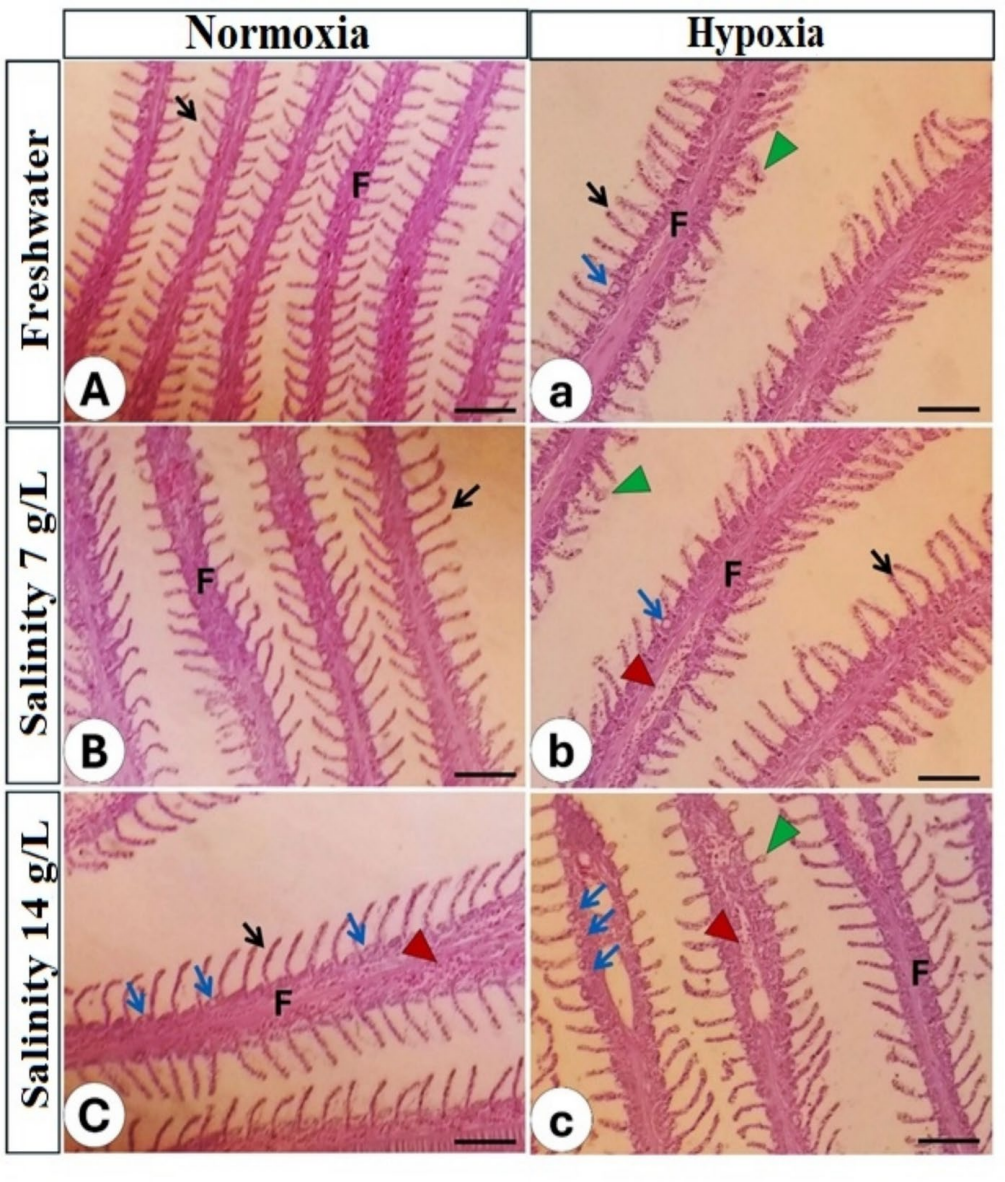
Histopathology of gills of Nile tilapia reared in different water salinity and two oxygen levels. The left column refers to normal oxygen and different salinity levels (A–C), while the right column refers to low oxygen and different water salinity (a–c). F; primary lamellae, black arrow; secondary lamellae, blue arrow; chloride cells, green arrowhead; telangiectasia, red arrowhead; vascular congestion. Stain H&E. Bar = 100 µm
c The liver:
  The histopathological structure of *O. niloticus* liver reared in freshwater under normoxia revealed typical hepatic cells, hepatic vessels, and pancreatic acini (Fig. 7A). Upon increasing salinity, vascular dilatation and congestion of both the central vein and the blood sinusoid are observed (Fig. 7B, C)**.** This hazardous effect was intensified by the interaction with hypoxia, which induced vascular congestion even at lower salinities (Fig. 7a). The most severe pathological changes, including severe degeneration, vacuolation, and nuclear pyknosis of hepatocytes and pancreatic acinar cells, were distinctly observed in fish subjected to the combined stress of hypoxia and high salinity (14 g/L) (Fig. 7b, c; Table 9).
  **Fig. 7:**
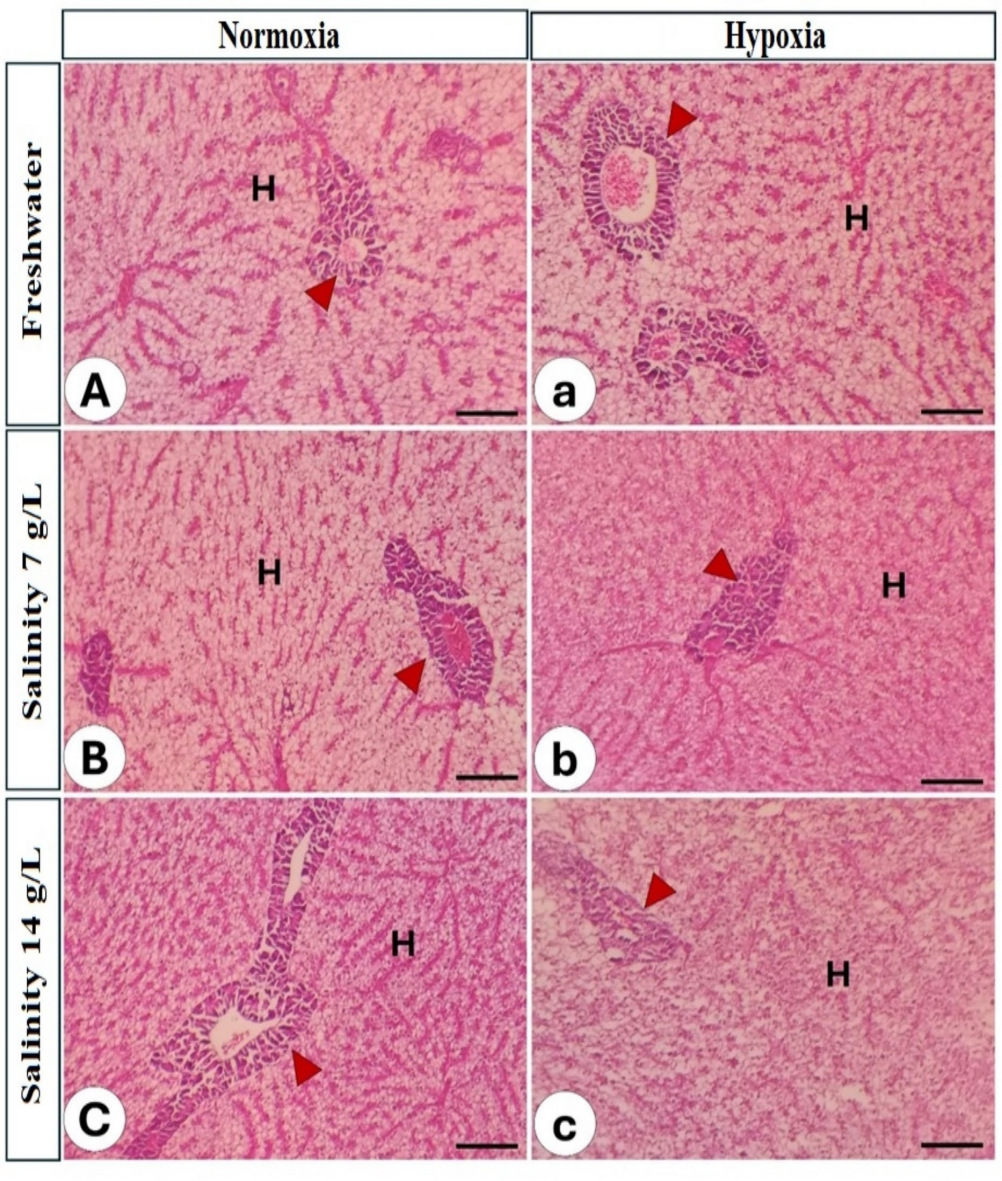
Histopathology of liver in Nile tilapia reared in different water salinity and two oxygen levels. The left column refers to normal oxygen and different salinity levels (A–C), while the right column refers to low oxygen and different water salinity (a–c). H; hepatocytes, red arrowhead: pancreatic acini, Stain H&E. Bar = 100 µm

## Discussion

### Water quality

Our findings revealed that all water quality measurements are suitable for aquaculture, according to Boyd and Tucker (2012). Only salinity and its derivatives (TDS & conductivity) and oxygen differed significantly, as the experimental design protocol acknowledged. Various finfish species’ growth behavior and production are contingent upon optimal water salinity (McCarthy et al. 2020). Since fish are euryhaline creatures, their normal growth and production depend on the water’s ideal salinity (Chang et al. 2021a; Mozanzadeh et al. 2021).

### Growth performance

In the current investigation, fish growth performance decreased with increased salinity under hypoxia, while the best growth performance was at freshwater treatment under normoxia. These results were due to an inconsistent osmoregulation function caused by excessive salinity, which leads to the consumption of high energy, resulting in the decline of the metabolic and growth rates (Bœuf and Payan 2001; Chourasia et al. 2018; Herrera et al. 2009), disrupted physiological functions and heightened oxidative stress caused by increased salinity (Dawood et al. 2022a). Furthermore, the fish’s metabolic rate is also strongly affected by the water’s dissolved oxygen content (Bulbul Ali and Mishra 2022). Furthermore, when dissolved oxygen concentration diminishes, feeding activities and other physiological processes decline; thus, the growth rate diminishes, and the fish cannot assimilate nutrients (Bulbul Ali and Mishra 2022; McNicholl et al. 2021). Fish minimize feed consumption and suspend growing when water oxygen saturation drops below 70% (Jobling 1993). During hypoxia, feed consumption and growth rate are markedly reduced compared to normoxia (Abdel-Tawwab et al. 2015, 2014).

On the other hand, diminished growth performance is likely ascribed to reduced feed utilization and impaired digestion within the intestinal tract of fish (Chourasia et al. 2018). According to the present evaluation, both stressors, hypersalinity and hypoxia, together have reduced Nile tilapia’s feed consumption. Since both hypoxia and increased salinity reduced feed intake, their combined effect resulted in the lowest feed intake under concurrent exposure, highlighting a synergistic negative impact of these stressors on feeding behavior. Indeed, hypersalinity lowers feed intake and utilization, hence lowering digestibility and metabolic activity (Rahmah et al. 2020). Moreover, fish under hypoxia had diminished feed intake and growth due to lower appetite and digestibility (Gan et al. 2013; Tran‐Duy et al. 2012). Furthermore, under hypoxic conditions, fish cannot utilize their feed (Nehemia et al. 2012). Thus, in the current study, it can be concluded that higher growth performance and feed utilization under normoxic conditions primarily stemmed from improved feed intake and nutrient digestibility. Optimal dissolved oxygen levels enhance mobility and digestion (Bulbul Ali and Mishra 2022).

Comparable outcomes had been reported with striped bass, *Morone saxatilis* (Brandt et al. 2009); big sea bass, *Micropterus salmoides* (Pichavant et al. 2001); common carp, *C. carpio* (Bernier et al. 2012); Atlantic salmon (Remen et al. 2012); turbot, *Scophthalmus maximus* (Ruyet et al. 2003); silver salmon, *O. kisutch* (Brett and Blackburn 1981); and Nile tilapia, *Oreochromis niloticus* (Abdel-Tawwab et al. 2015, 2014; Bergstedt et al. 2021; Dawood et al. 2021; Li et al. 2018; Tran‐Duy et al. 2012). In this regard, Bulbul Ali and Mishra (2022) reported that fish growth might be reduced even after short hypoxic periods.

### Survival

In this study, salinity stress did not affect fish survival, showing great endurance, potentially due to tropical freshwater species ingesting air. Some fish can endure 30–34 ppt salinity, indicating Nile tilapia may be tolerant (El-Leithy et al. 2019). However, Kang’ombe and Brown (2008) observed that Nile tilapia survival rates changed markedly with salinity, possibly because of their modest tolerance and death over 20 g/L (Kirk 1972). Nile tilapia can survive above 4 g/L, according to Iqbal et al. (2012). Depending on bodily fluid function, fish may tolerate varying salinities for short durations (Holliday and Jones 1967; Kurata 1959).

On the other hand, Nile tilapia’s resistance to low oxygen levels (as low as 1.0 mg/L DO_2_) in the current study may enable it to consume ambient oxygen sparingly (Abdel-Tawwab et al. 2015, 2014; Ross 2000). They survive hypoxia by entering metabolic depression and performing aquatic surface respiration (Obirikorang et al. 2020; Bergstedt et al. 2021).

### Digestive enzymes

The catalytic efficacy of digestive enzymes is influenced by several ecological factors, including temperature, pH, salinity, and dissolved oxygen levels (Hu et al. 2024; Jiang et al. 2020; Luo et al. 2015). The adverse influence of increased salinity on digestive enzyme activity in the current investigation was ascribed to fluctuations in pH and ion levels within the digestive tract due to salt consumption or water loss during osmoregulatory operations, which inhibit enzymatic activities (Nguyen et al. 2021). Moreover, salinity influences the osmotic pressure of the aquatic animal body, thus influencing gastrointestinal digestive enzyme activity (Liu et al. 2024; Monier et al. 2025). Salinity variations may also affect the water’s ion concentration, which can impact the performance of digestive enzymes. Earlier investigations have concluded that salinity significantly influences the digestive enzymes of aquatic animals, therefore influencing their growth and maturation (Monier et al. 2025; Pujante et al. 2018; Zhang et al. 2023). In this regard, Monier et al. (2025) observed that increasing salinity negatively decreased the protease, α-amylase, and lipase activity in the Nile tilapia intestine.

On the other hand, our investigation revealed that hypoxia also negatively impacted digestive enzyme activity, which may be attributed to the drop in energy accessible for physiological processes during hypoxic stress, resulting in the suppression of general metabolic activity (Gu et al. 2019; Yang et al. 2021). The aerobic breakdown within the fish’s body is diminished in hypoxic conditions. Consequently, the anaerobic glycolysis is increased, which alters the activity of the associated enzymes, thereby decreasing the energy necessary for survival and jeopardizing the fish’s health (Hu and Lin 1999). Furthermore, hypoxia-induced anxiety may lead to oxidative stress and an energy deficit in gastrointestinal cells, resulting from a diminished digestion process and lower energy consumption (Amado and Monserrat 2010; Prieto et al. 2007). Hypoxia stress is believed to have reduced the growth rate of cobia juveniles, resulting in noticeable weight loss (Weizheng et al. 2021); this phenomenon may be attributed to diminished activities of digestive enzymes, leading to impaired digestion and absorption of feed. Resistance to hypoxic stressors varies by species and is influenced by their lifespan phases (Vaquer-Sunyer and Duarte 2008). Shi et al. (2019) demonstrated that exposure to low oxygen levels (3.0 mg/L DO_2_) resulted in substantially decreased levels of pepsin, lipase, and α-amylase in sweetfish (*Plecoglossus altivelis*) compared to the normoxic control group. Hypoxia anxiety reduced the production of digestive enzymes in rainbow trout (*Oncorhynchus mykiss*) (Zhao et al. 2007) and juvenile cobia (*Rachycentron canadum*) (Yang et al. 2021).

### Hematological parameter

In the current investigation, the RBCs count in all salinities was substantially decreased compared to the freshwater group (0‰), which may have occurred from osmotic alterations caused by salinity resulting from ion loss from the plasma (Alwan et al. 2009; Uddin et al. 2023). After being subjected to sub-lethal osmotic tension, the decreased RBC number suggests a diminished capacity for blood oxygen transport. Das et al. (2006) found that subjection to hypersalinity led to the distortion and decomposition of specific RBC cells in Silver barb, impairing blood oxygen-transport potential. This impairment can be mitigated by increasing oxygen attraction and hemoglobin potential, enhancing the formation of red blood cells. In the current investigation, the hemoglobin levels in the blood of Nile tilapia were markedly reduced at elevated salinities of 7 and 14‰ compared to those at 0‰, which may be attributed to the disturbance of hematopoietic roles resulting from the tension induced by hypersalinity (Elarabany et al. 2017; Islam et al. 2020b; Uddin et al. 2023). The alterations in hematological indicators due to hypersalinity vary and are contingent upon specific species’ acclimatization and adaptation abilities, and the magnitude of the salinity shift. In a parallel study, Elarabany et al. (2017) indicated that hemoglobin level was drastically decreased in *O. niloticus* subjected to salinities of 8 and 12‰. The current research suggests that the reduction of Hb may be linked to osmoregulatory failure in aquatic animals subjected to elevated salinities (Soltanian et al. 2016; Uddin et al. 2023).

Contrary to the influence of salinity, in the current experiment, red blood cells, hemoglobin, and hematocrit levels rose in Nile tilapia blood during hypoxia, which was attributed to the fish adapting to the hypoxic conditions by increasing circulation and red blood cell quantity. Under hypoxic tension, fish typically reduce oxygen intake by decreasing movement and enhancing oxygen-carrying capacity via elevated RBC and Hb concentrations (Abdel-Tawwab et al. 2019; Xia et al. 2016). Numerous investigations have demonstrated that fish subjected to hypoxic conditions displayed rapid elevations in RBCs, hemoglobin, and/or hematocrit (Abdel-Tawwab et al. 2015, 2014; Affonso et al. 2002; Ruiz et al. 2024; Wells and Baldwin 2006). Under normoxia (85.4% DO_2_), gilthead seabream exhibited substantially decreased hematocrit concentrations compared to those subjected to hypoxia (Araújo‐Luna et al., 2018). The rise in red blood cell count under hypoxia might be caused by the contraction of the fish spleen, which releases a significant quantity of red blood cells into the circulatory system to improve oxygen transport (Douxfils et al. 2012). Ruiz et al. (2024) exposed rainbow trout to 2 mg/L DO_2_ for 1 h, after which the fish were set to heal for 1 h. Their findings revealed a sequence of adaptation represented by values of Hb, Hct, and MCV, suggesting an exceptional capability of rainbow trout to withstand this kind of frequent hypoxic condition, proving that the fish could have some healing time following the exposures. However, in the current research, the group exposed to hypoxia with salinity 7 (HS7) showed a marked increase in RBCs and hemoglobin levels compared to normoxia with salinity 7 (NS7), indicating that under moderate salinity, the hypoxic condition has a more pronounced effect on RBC and hemoglobin production, suggesting that hypoxia might have a stronger impact on blood cell production than salinity at lower salinity levels (Abdel-Tawwab et al. 2019; Ali et al. 2024; Uddin et al. 2023; Wu et al. 2024).

The count of white blood cells in fish is an accurate biomarker for assessing physiological standing (Paul et al. 2023; Svobodová et al. 2001). It likewise serves a vital role in rapidly removing cellular fragments resulting from tissue necrosis (John 2007; Uddin et al. 2023). The current study indicates that elevated WBC counts in fish at higher salinities may result from increased antibody formation, facilitating recovery and survival under stressful conditions (Uddin et al. 2023). In Uddin et al. (2023), freshwater Gourami (*Trichogaster fasciata*) subjected to salinity stress for 30 days showed notably increased WBC counts, which would have resulted from the disruptions in acid–base equilibrium, respiratory homeostasis, and ionic control. On the contrary, fish’s white blood cell (WBC) counts are elevated under hypoxic conditions compared to normoxic conditions in the present investigation, which may be attributed to elevated leukocytosis from the immunological response to stressful circumstances. The immune system’s response to stress increases the number of white blood cells by activating the leukopoietic process and promoting the release of leukocytes into the bloodstream (Uddin et al. 2023). Numerous research findings indicate a substantial rise in white blood cell counts in fish under stressful circumstances, corroborating the current study (Akinrotimi and Amachree 2016; Geetha 2014; Uddin et al. 2023).

### Biochemical indices

The biochemical and physiological state of cultivated fish is crucial for assessing their health. Variations in these parameters may indicate adverse ecological circumstances or the presence of stressors, including feed limitations, overcrowding, toxic substances, excess organic compounds, and standard aquaculture practices (Abdel-Tawwab et al. 2019).

Cortisol and glucose are biomarkers associated with stress that may indicate stressful conditions (Albaqami and Monier 2025; Bertotto et al. 2010). In fish, cortisol patterns under stress are marked by a rapid initial increase, peaking within minutes to an hour depending followed by a gradual decline depending on the fish species and age, and even environmental conditions like temperature or the type and severity of the stressor (Barton 2002; Iwama et al. 2004; Kalamarz-Kubiak 2018). In the current study, the elevation in plasma cortisol and glucose levels in fish stressed by hypoxia and high salinity, compared to the non-stressed group (normoxia at freshwater), may be attributed to the activation of the fish stress response system. Under such unfavorable conditions, the brain–sympathetic–chromaffin (BSC) axis is first activated, leading to the rapid secretion of catecholamines (adrenaline and noradrenaline) from chromaffin cells into the bloodstream. Subsequently, the hypothalamus–pituitary–interrenal (HPI) axis is activated, stimulating cortisol release from interrenal cells (Barton 2002; Iwama et al. 2004; Kalamarz-Kubiak 2018). Those increase cortisol levels in the bloodstream, which affect carbohydrate metabolism, enhancing glucose production via gluconeogenesis and glycogenolysis to meet increased energy requirements (Barton 2002; Iwama et al. 2004; Kalamarz-Kubiak 2018). The present investigation also suggests that elevated blood glucose levels in fish reared in high salinity may result from a faster frequency of glucose delivery from the liver to the bloodstream, needed by heightened energy demands for quick and irregular movements (Albaqami and Monier 2025; Islam et al. 2020a; Uddin et al. 2023). Moreover, fish frequently secrete plasma glucose to get energy to alleviate the detrimental effects caused by stress (Wendelaar Bonga 1997). These findings are comparable with our previous finding in which we observed increased cortisol and glucose secretion after subjecting Nile tilapia to thermal or hypersalinity stress (Albaqami and Monier 2025). Additionally, catfish (*P. hypophthalmus*) that resided in high-salinity and elevated temperature habitats had higher concentrations of cortisol and glucose, according to Phuc et al. (2017). Ni et al. (2014) and Sheng et al. (2019) stated that Amur sturgeon (*Acipenser schrenckii*) and GIFT tilapia juveniles, respectively, consistently increased cortisol levels when subjected to hypoxia.

AST and ALT are biomarkers of health and cellular membrane integrity (Costas et al. 2011), indicating liver condition (Ghodrati et al. 2021), signifying metabolic disorders and indications of hepatic injury and failure (Albaqami and Monier 2025; Gao et al. 2022; Guo et al. 2023; Monier et al. 2025). The current study demonstrated that blood ALT and AST levels in the hypoxia and hypersalinity groups were significantly higher than those in the normoxia group with freshwater. These indicate that hypoxia and/or salinity stress resulted in tissue degradation in Nile tilapia, causing hepatic injury and potential liver dysfunction due to oxidative strain altering metabolite and liver enzyme secretions, increasing the permeability of the liver cell membrane and permitting ALT and AST to reach the circulation in high levels (Albaqami and Monier 2025; Chang et al. 2021b; Ghelichpour et al. 2020). Increased salinity (10 and 20 g/L) significantly elevated ALT levels in Nile tilapia circulation (Dawood et al. 2021).

Fish stressed by hypoxia and higher salinity levels exhibited high levels of T-CHO and TG; this may be linked to the disturbance in lipid metabolism, which leads to hyperlipidemia, hypercholesterolemia, immediate atherosclerosis, and an accumulation of high fat (Albaqami and Monier 2025; Javed and Usmani 2015).

Plasma proteins, albumin, and globulin have crucial functions in innate immunity responses and are well-recognized as reliable indices of humoral immune responses (Hoseinifar et al. 2018). In this study, the observed reduction in plasma protein under hypoxia and increased salinity can be attributed to the degradation of proteins into carbon compounds and amino acids, which serve as energy sources to mitigate stress (Albaqami and Monier 2025; Monier et al. 2025). Initially, fish rely on glucose and triglycerides as energy sources during hypoxic stress, while serum protein is reduced over time. Proteins take over as the primary supplier of metabolic energy under sudden hypoxia as soon as all other alternatives have been exhausted, based on research on yellow croaker (*Larimichthys crocea*) and sockeye salmon (*Oncorhynchus nerka*) (Shahjahan et al. 2022). The reduction in total protein proposes that protein is used in energy generation (Albaqami and Monier 2025; Ding et al. 2020; Monier et al. 2025).

Albumin plays a vital role in maintaining stable blood osmotic pressure and participates in various functions, including nutrient transport, coagulation, anticoagulation, and the repair and renewal of hepatocytes. The primary role is maintaining a stable chemical environment within the body (Sheng et al. 2019). The results of our study demonstrate a significant reduction in blood albumin levels in fish following exposure to increased salinity and hypoxia, consistent with the findings of Albaqami and Monier (2025), Guo et al. (2023), and Monier et al. (2025). The decline in albumin levels may be due to stress from excessive salinity stress and hypoxia. The body presumably uses albumin to supply energy and maintain plasma colloid osmotic pressure, causing its reduction. The fish’s liver failed due to high salinity and hypoxia stress as the exposure time grew. This liver failure affected protein metabolism and lowered serum albumin (Albaqami and Monier 2025; Guo et al. 2023; Monier et al. 2025).

### Antioxidant and lipid peroxidation

Since fish consume oxygen, dissolved oxygen is a significant ecological element influencing their antioxidant defense mechanisms and physiological processes (Abdel-Tawwab et al. 2019). Oxidative stress represents a significant detrimental operation induced by adverse conditions impacting aquatic organisms (Limbu et al. 2018). SOD and CAT act on superoxide free radical and hydrogen peroxide, respectively; meanwhile, GPx is crucial in the process of mitigating the oxidative damage (Kehrer 1993). MDA is the end product of fatty acid peroxidation, and its concentration can be measured to determine the level of lipid peroxidation. It is generally used as an indicator of oxidative stress (Del Rio et al. 2005).

The present examination indicates that reducing antioxidant enzymes in fish subjected to hypoxia and increased salinity signifies oxidative impairment in the hepatic tissue resulting from such stressors. Stress prompts fish to start an antioxidant response that targets eliminating reactive oxygen species like H_2_O_2_ and O_2_-, thereby mitigating oxidative damage (Qi et al. 2020; Tang et al. 2023). Additionally, the current study indicates that elevated hepatic MDA levels in Nile tilapia subjected to hypoxia and salt stress signify lipid peroxidation resulting from stress. When fish are subjected to stressors, there is a substantial rise in MDA levels in both their blood and tissues (Albaqami and Monier 2025; Monier et al. 2025).

### Immunity

Alterations in environmental factors, including salinity, dissolved oxygen, and various stressors, may induce adaptations in physiological mechanisms to maintain homeostasis, potentially affecting standard biological functions, such as immune function and activity (Albaqami and Monier 2025; El-Leithy et al. 2019). Nonetheless, cellular immunity responses assessed fish’s immunological health after experiencing stress or adverse circumstances (Ortuño et al. 2003, 2001).

The present research suggests that the suppression of immune response attributed to increasing salinity and hypoxia could result from the impairment of osmoregulatory operations caused by higher salinity levels, as indicated by previous studies (Soltanian et al. 2016; Usha 2011), along with the energetic demands associated with chronic stressful responses (Abdel-Tawwab et al. 2019; Dawood et al. 2022a). Indeed, the fish’s energy expenditure will be reduced if a portion of it is allocated to stressors, which will result in an energy reduction accessible for additional biological processes, such as immune function (Abdel-Tawwab et al. 2019; Douxfils et al. 2012; Segner et al. 2012). Hypoxia has been demonstrated to alter aquatic creatures’ adaptive and innate immunity responses (Abdel-Tawwab et al. 2019; Kvamme et al. 2013). Previous studies have indicated substantial mortality due to streptococcal contamination in Nile tilapia when exposed to hypoxia stress (Evans et al. 2005). Long-term stress may suppress the immune system, reducing the body’s ability to fight off illnesses (Magnadottir 2010).

Salinity is a critical abiotic factor that substantially influences the growth, metabolism, immunity, and survival of aquatic animals in natural and cultural circumstances (Jeffries et al. 2019; Kültz 2015). Specifically, changes in salinity impose osmotic stress on the animal, forcing it to expend significant metabolic energy on osmoregulation (Baldisserotto et al.  2007; Cai et al. 2020). This energy reallocation can come at the expense of the immune system, leading to a suppressed immune response and increased susceptibility to diseases (Birrer et al. 2012; Cai et al. 2020). Furthermore, extreme or fluctuating salinity levels can elevate stress hormones like cortisol, which are known to have immunosuppressive effects on the fish’s immune system (Tort 2011).

Under normoxic conditions, immune parameters such as lysozyme, respiratory burst, phagocytic activity, and total IgM were highest in freshwater (NS0) and slightly declined with increasing salinity (NS7 and NS14), indicating mild immunosuppression linked to osmotic adjustments.

Respiratory burst and lysozyme activities are vital in innate immunity systems (Ellis 1990; Grinde 1989). Lysozyme is necessary to break down the cell walls of harmful Gram-positive or negative bacteria (Ellis 1990; Monier et al. 2025; Saurabh and Sahoo 2008). Meanwhile, respiratory burst activity describes the rapid intensification in the generation of ROS that occurs during the phagocytosis of microorganisms. It is essential in innate immunity, aiding phagocytic cells in eradicating pathogens (Secombes and Fletcher 1992). Phagocytic cells are essential to aquatic animals’ innate immunity defense since they have mechanisms to defend against pathogens (Secombes and Fletcher 1992). However, IgM has vital functions in the immunity of both innate and adaptive aquatic animals. In the present investigation, decreased respiratory burst, lysozyme activity, and phagocytosis may be due to the activation of complements that attack and lyse pathogens (Boshra et al. 2004; Cooper 1985). Moreover, immunoglobulin assists in the aggregation of pathogens for phagocytosis, pathogen removal, and cell degradation (Ye et al. 2013).

In the present investigation, fish plasma respiratory burst and lysozyme values decreased with increased salinity under hypoxia, which may have been attributed to changes in dissolved oxygen levels that have modulated fish’s innate immunity responses (Abdel-Tawwab et al. 2019, 2015, 2014). Abdel-Tawwab et al. (2014) discovered that the innate immunity relies on dissolved oxygen. Unfavorable water quality resulting from human activities or detrimental environmental conditions, such as hypoxia and salinity, can impair immunity, leading to a decreased tolerance to infection by pathogens (Di Marco et al. 2008). Abdel-Tawwab et al. (2015, 2014) investigated the impact of varying oxygen concentrations on the innate immunity of Nile tilapia infected with the infective bacteria *A. hydrophila*. They concluded that when dissolved oxygen levels dropped, fish immunity correspondingly decreased and fish susceptibility to *A. hydrophila* contagion.

### Histopathology

The interruption of the histological structures in the intestine, liver, and gills tissues in the present study with increased salinity under hypoxic conditions agrees with the previous research on Nile tilapia, which observed increased hyperplasia, degeneration, and sloughing in Nile tilapia intestine with raised salinity (Albaqami and Monier 2025; Dawood et al. 2023, 2021; Tran-Ngoc et al. 2017).

Hypoxia and salinity stress cause considerable structural alterations in the fish intestine, impacting nutrient intake and immunological responses. In the present assessment, hypoxia reduces villus height and crypt depth, while concurrently enhancing goblet cell proliferation, presumably as a protective response to chronic stress (Kim 2017; Soares et al. 2022). On the other hand, the lamina propria thickens under both normoxic and hypoxic conditions, showing a gradual increase with rising salinity levels, indicating an inflammatory response to stress (Tran-Ngoc et al. 2016). Additionally, in the current investigation, salinity stress equally boosts goblet cell density, likely promoting osmoregulation (Rodríguez et al. 2005), while also resulting in mucosal damage and shortened villi (Soares et al. 2022; Sumon et al. 2022). The increase in goblet cell numbers reflects the adaptation response to saline conditions, which agrees with the findings in Mozambique tilapia (Li et al. 2014) and Nile tilapia (Soares et al. 2022). These alterations suggest that salinity stress may impair epithelial barrier function, as demonstrated in hypoxic conditions (Tran-Ngoc et al. 2016). The morphological alterations underscore the adaptive mechanisms of the intestine in response to environmental stressors.

In the current investigation, the simultaneous exposure to hypoxia and elevated salinity resulted in significant deterioration accompanied by catarrhal exudate in the majority of intestinal structures, along with hyperplasia in the tunica muscularis and infiltration of inflammatory cells, which agreed with the previous investigations (Albaqami and Monier 2025; Dawood et al. 2023, 2021). Intestinal impairment was also seen in Nile tilapia subjected to either temperature or salt (Albaqami and Monier 2025), as well as the combined effects of ammonia and salinity (Dawood et al. 2023). Oxidative stress-induced inflammation is likely the primary cause of intestinal impairment in Nile tilapia, which is subjected to hypoxia and elevated salinity.

In the present observations, the histopathological status of the Nile tilapia liver displayed a significant impact of salinity and hypoxia stress on hepatic integrity and function. Under normoxic conditions, the freshwater-treated fish’s liver had a typical structure characterized by healthy hepatocytes, intact hepatic arteries, and well-defined pancreatic acini, reflecting an ideal physiological state (Dawood et al. 2021). These outcomes are consistent with previous research demonstrating that tilapia can maintain appropriate hepatic metabolism and cellular homeostasis in fully oxygenated freshwater environments (Li et al. 2024). Nonetheless, elevated salinity in the current study resulted in notable pathological changes, including vascular dilatation and congestion of the central vein and blood sinusoids. These pathological changes indicate increased circulatory demands and hemodynamic anxiety resulting from osmoregulatory modifications necessary to maintain ionic equilibrium (Albaqami and Monier 2025; Dawood et al. 2022a). Additionally, elevated vascular congestion is often associated with inflammation induced by osmotic stress, which can negatively impact proper hepatic function (Esam et al. 2022).

In the present study, the simultaneous exposure to elevated salinity and hypoxia intensified hepatic injury, resulting in hepatocyte degeneration, vacuolation, and nuclear pyknosis. These findings suggested cellular distress, metabolic instability, and apoptosis, which may be attributed to oxidative stress and energy deprivation (Elbialy et al. 2021). These characteristics may explain disturbances in antioxidant activity and lipid peroxidation, as well as elevated concentrations of ALT and AST, and decreased total protein levels in the bloodstream. Our findings align with Albaqami and Monier (2025) and Dawood et al. (2023, 2021), who documented compromised liver histological characteristics resulting from salinity stress.

Hepatic vacuolation is often associated with excessive lipid buildup, glycogen depletion, or toxin accumulation under stress, resulting in diminished metabolic efficiency (Dawood et al. 2022c). Nuclear pyknosis, indicative of cell death, implies irreversible hepatocellular damage resulting from hypoxia-induced mitochondrial malfunction and oxidative stress (Dawood et al. 2022c). According to our hepatic histopathological score results, hypoxia and salinity stress exacerbate hepatic injury, impairing its function (Refaey et al. 2025). This is crucial for the proper functioning of the immune system, metabolism, and detoxification. The health and growth of fish may be considerably affected by their deterioration (Motamedi-Tehrani et al. 2025).

Gill histopathology is a reliable biomarker for environmental stress (Hasan et al. 2022). The histopathological changes in the gills of Nile tilapia due to changes in environmental salinity and oxygen levels are of significant interest due to their implications for aquaculture, water quality management, and fish health (Dawood et al. 2021; Mohamed et al. 2021). The present study demonstrates that the gill structure remains intact, featuring well-preserved primary and secondary filaments and maintaining the gill epithelium’s structural integrity under optimal conditions (freshwater and normoxia) (Hassan et al. 2013). The normal gill epithelium facilitates effective ion transport, osmoregulation, and gas exchange (Albaqami and Monier 2025; Dawood et al. 2023, 2021; Mohamed et al. 2021). In the present research, slight degeneration of the primary filament begins at a salinity of 7 g/L, indicating an early adaptive response to osmotic stress (Rahmati et al. 2022). Moreover, vascular congestion occurs in the primary filaments by increased salinity to 14 g/L, suggesting increased stress on the gill vasculature due to osmotic imbalance and higher metabolic demand (Mohamed et al. 2021). Long-term exposure to high salinity (14 g/L) results in severe structural disruptions, including epithelial lifting, hyperplasia, and lamellar fusion, which impair respiratory efficiency and ion balance (Dawood et al. 2021). The present study suggests that hypoxia exacerbates the adverse effects of salinity stress by constricting gill blood vessels due to circulatory stress and a decreased oxygen-carrying capability (Islam et al. 2022).

Meanwhile, the increased density of chloride cells suggests an effort to enhance ion transport as a response to osmotic stress (Xing et al. 2022). Telangiectasia, characterized by the dilation of secondary filaments, is likely a compensatory mechanism to optimize oxygen uptake in a hypoxic environment (Dawood et al. 2022b). The deterioration of the lining epithelium serves as a significant indicator of prolonged stress, which may lead to impaired respiratory efficiency and elevated vulnerability to disease (Dawood et al. 2023).

## Conclusion

The current investigation revealed the substantial interaction effects of hypoxia and increased salinity on Nile tilapia’s growth performance, welfare, immunological, and histopathological status. Hypoxia and increased salinity negatively impacted growth performance and feed efficiency, disrupted digestive enzyme activity, and altered hematological indicators, indicating physiological and metabolic stress. Moreover, hypoxic and greater salinity conditions caused oxidative stress, leading to elevated lipid peroxidation and reduced antioxidant enzyme activity, thereby increasing tissue damage. Moreover, the immunological reaction was suppressed under hypoxia and exhibited an apparent reduction in innate immunity with increasing salt concentration. Histopathological alteration in the liver, gills, and intestine revealed substantial structural damage, reflecting the adverse effects of environmental stresses on fish health.

Our results highlight the need to enhance aquaculture techniques to lower unfavorable consequences, as they reveal the vulnerability of Nile tilapia to combined hypoxia and salinity stress. Strategies such as water management, controlled aeration, and nutritional approaches and additives should be studied to increase fish tolerance to environmental fluctuations driven by climate change, which could help create effective and sustainable aquaculture strategies.

## Data Availability

Data will be made available on request.
